# Factors influencing access to specialised haematology units during acute myeloblastic leukaemia patient care: A population‐based study in France

**DOI:** 10.1002/cam4.5645

**Published:** 2023-01-29

**Authors:** Kueshivi Midodji Atsou, Bernard Rachet, Edouard Cornet, Marie‐Lorraine Chretien, Cédric Rossi, Laurent Remontet, Laurent Roche, Roch Giorgi, Sophie Gauthier, Stéphanie Girard, Johann Böckle, Stéphane Kroudia Wasse, Helene Rachou, Laila Bouzid, Jean‐Marc Poncet, Sébastien Orazio, Alain Monnereau, Xavier Troussard, Morgane Mounier, Marc Maynadie

**Affiliations:** ^1^ Registre des Hémopathies Malignes de la Côte‐d'Or CHU de Dijon Bourgogne Dijon France; ^2^ UMR INSERM 1231 Université Bourgogne Franche‐Comté Dijon France; ^3^ Inequalities in Cancer Outcomes Network, Department of Non‐communicable Disease Epidemiology, Faculty of Epidemiology and Population Health London School of Hygiene & Tropical Medicine London UK; ^4^ Registre régional des hémopathies malignes de Basse‐Normandie CHU Caen‐Normandie Caen France; ^5^ CHU Dijon Bourgogne Service d'Hématologie Clinique Dijon France; ^6^ Pôle Santé Publique, Service de Biostatistique ‐ Bio‐informatique Hospices Civils de Lyon Lyon France; ^7^ UMR 5558, Laboratoire de Biométrie et Biologie Évolutive, Équipe Biostatistique‐Santé Université de Lyon, Université Lyon 1, CNRS Villeurbanne France; ^8^ SESSTIM, Sciences Économiques & Sociales de la Santé & Traitement de l'Information Médicale, ISSPAM, Hop Timone, BioSTIC, Biostatistique et Technologies de l'Information et de la, Communication Aix Marseille Univ, APHM, INSERM, IRD Marseille France; ^9^ Registre des Hémopathies Malignes de Gironde Institut Bergonié Bordeaux France; ^10^ EPICENE Team, Inserm U1219, Bordeaux Population Health University of Bordeaux Bordeaux France

**Keywords:** acute myeloblastic leukaemia, care pathways, logistic regression, population‐based data, specialised haematology unit access

## Abstract

**Background:**

The excess mortality observed in Acute Myeloblastic Leukaemia (AML) patients, partly attributed to unequal access to curative treatments, could be linked to care pathways.

**Methods:**

We included 1039 AML incident cases diagnosed between 2012–2016 from the 3 French blood cancer registries (3,625,400 inhabitants). We describe patients according to age, the medical entry unit and access to the specialised haematology unit (SHU) during follow‐up. Multivariate logistic regression model was done to determine the association between covariables and access to SHU. A total of 713 patients (69%) had access to SHU during care.

**Results:**

The most common care pathway concerned referral from the general practitioner to SHU, *n* = 459(44%). The univariate analysis observed a downward trend for the most deprived patients. Patients who consulted in SHU were younger (66 years vs. 83, *p* < 0.001), and 92% had access to cytogenetic analysis (vs. 54%, *p* < 0.001). They also had less poor prognosis AML‐subtypes (AML‐MRC, t‐AML/MDS and AML‐NOS) (38% vs. 69%); 77% with de novo AML (vs. 67%, *p* < 0.003)], more favourable cytogenetic prognostic status (23% vs. 6%, *p* < 0.001), less comorbidities (no comorbidity = 55% vs. 34%, *p* < 0.001) and treatments proposed were curative 68% (vs. 5.3%, *p* < 0.001). Factors limiting access to SHU were age over 80 years (OR, 0.14; 95% CI, 0.04–0.38), severe comorbidities (OR, 0.39; 95% CI, 0.21–0.69), emergency unit referral (OR, 0.28; 95% CI, 0.18–0.44) and non‐SHU referral (OR, 0.12; 95% CI, 0.07–0.18). Consultation in an academic hospital increased access to SHU by 8.87 times (95% CI, 5.64–14.2).

**Conclusion:**

The high proportion of access to cytogenetic testing and curative treatment among patients admitted to SHU, and the importance of early treatment in AML underlines the importance of access to SHU for both diagnosis and treatment.

## Graphical abstract

In this study, we show for the first time that well‐known clinical and biological prognostic factors limit the access of AML patients to a specialised haematology unit, whereas this consultation strongly influences the access to cytogenetic analyses and curative treatments.

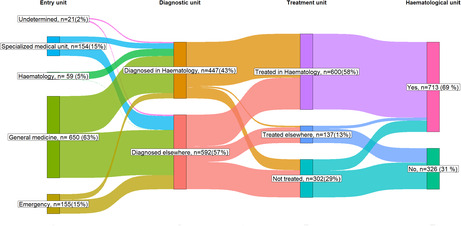

## 1 INTRODUCTION

Acute Myeloblastic Leukaemia (AML), although a rare disease of the elderly, accounts for 80% of acute leukaemia in adults.^1^ With a 5‐year net survival of 27%, AML has a very poor prognosis,^2^ except for patients with a t(15;17) translocation who benefit from a specific treatment.^3^

Over the last few decades, cytogenetic and molecular profiling tools for AML have significantly improved^4^, ^5^ our understanding of the AML molecular landscape which in turn has allowed improved classification of AML. These advances have also facilitated the development of new molecules targeting specific mutations such as those targeting the FLT3 or IDH genes respectively.^6^, ^7^ These advances have also contributed to improved stratification of AML patients into prognostic groups that allow to better adapt treatments and to treat a higher number of patients.^8^, ^9^, ^10^ Despite this, the therapeutic management scheme, particularly in the general population, remains similar for most subtypes and is based on a combination of anthracycline and cytarabine,^11^ except for AML subtypes with t(15;17).^12^ However, a slight increase in net survival has been observed in AML patients (+14% net survival at 1 year, and +15% net survival at 5 years, for cases diagnosed between 1990–2015), but these patterns differ among patients, notably according to age.^2^ These differences could be explained by biological factors intrinsic to the disease and to patient clinical characteristics such as the presence of comorbidities which have an influence on patient eligibility for treatment.^8^

Differences in survival have also been attributed, at least in part, to unequal access to curative treatments, which in turn is potentially influenced by preventable, non‐biological factors associated with patient care pathways.^13^, ^14^, ^15^ As these treatments are mostly reserved to specialised care facilities, it is important to investigate the impact of the care pathway on treatment access and on patient survival. However, there are few data available in the literature on the AML patient care pathway.^16^, ^17^ A recent study has concluded that patients treated in academic institutions or high‐volume hospitals were better managed than those treated elsewhere.^18^ It was also found that patients treated in academic hospitals had better access to cytogenetic and molecular testing, to new drugs, a more likely inclusion in clinical trials and a greater probability of receiving a haematopoietic stem cell transplant. None of these studies, however, has assessed the real impact of access to a Specialised Haematology Unit (SHU) on the management of AML patients and potentially their survival, since widely available clinical trials do not optimally describe real‐life care.

Our study, which is part of the large French S‐LAM (Survival of Acute Myeloblastic Leukaemia patient) project on the management of all AML patients, aimed to describe, in a real‐life setting, the characteristics of the AML patient care pathway, including access to specialised care facilities in haematology and treatment management.

## 2 METHODS

### 2.1 Study design

The S‐LAM (Survival of Acute Myeloblastic Leukaemia) project is a retrospective longitudinal study including all incident AML cases diagnosed from 01 January 2012 to 31 December 2016 in the three French population‐based registries specialised in haematological malignancy (Côte‐d'Or, Basse‐Normandie and Gironde; around 3,625,400 inhabitants). For each patient, in addition to the core data (age, sex, place of residence, medical history, type of haematological cancer, medical follow‐up, treatment, sources of information, last date of follow‐up and vital status), we collected information on biological and molecular analyses, dates of occurrence of each event in the care pathway, including the various medical consultations and associated dates, and patient clinical evolution. The end point of patient follow‐up was set at 1 January 2021. The S‐LAM database was registered with the Commission Nationale de l'Informatique et des Libertés (CNIL) under number 921294. All data have been checked for integrity and quality.

### 2.2 Factors of interest

#### 2.2.1 Care pathway

We first defined seven care pathways (Emergency to SHU; Emergency to Non‐haematological unit; General Medicine to SHU; General Medicine to Non‐haematological unit; Specialised medical unit to SHU; Specialised medical unit to Non‐haematological unit and SHU only), by grouping patients according to their medical unit of admission and their diagnosis unit (Emergency, General medicine, Specialised medical unit and Specialised Haematology Unit). Then, for each of these groups, we distinguished between patients who completed their care pathway in a SHU from those who completed their follow‐up elsewhere (Appendix S1 ‐ Table 5).

We classified as academic facilities, the university hospitals and anti‐cancer centres. Non‐academic hospitals included peripheral hospitals, private health institutions of collective utility and medical practice offices.

#### 2.2.2 Tumours and patient characteristics

To describe our study population, we divided the patients into two groups, according to age at diagnosis: under and over 80 years old (y‐o) respectively, assuming that patients over 80 years of age are less likely to be treated.

Finally, we described patient characteristics according to the modalities of their access to haematological care facilities. For each modality, we report the distribution of cytogenetic and biomolecular prognostic markers, de novo or secondary AML profile, the Charlson Comorbidity Index (CCI) and the European Deprivation Index (EDI).^19^ We used the CCI as an indicator of patient comorbidities, while subtracting the weight of age in the calculation.^20^ Then, we grouped the CCI variable into three classes (0: No comorbidities, 1–2: low and mild comorbidities; ≥3: high comorbidities). Also, to be consistent with the study recruitment period, the European Leukaemia Network (ELN) 2016 working group classification was used to classify patient prognosis according to their cytogenetic status and molecular mutations.^21^ Based on treatment modalities, patients were grouped into three categories: untreated, non‐curative (supportive and palliative) and curative treatments (intensive chemotherapy).

#### 2.2.3 AML grouping

AML cases were categorised into six subtypes: AML‐RCA (AML with recurrent cytogenetics abnormalities (9865‐3, 9869‐3, 9871‐3, 9896‐3, 9897‐3, 9898‐3, 9877‐3); PML‐RARA (9866‐3); AML‐MRC (AML with multilineage‐related changes: 9895‐3, 9984‐3); t‐AML/MDS (therapy‐related AML/Myelodysplasia Syndrome: 9920‐3, 9987‐3); AML‐NOS (AML not otherwise specified: (9861‐3) and AML‐others (9931‐3, 9805‐3, 9806‐3, 9808‐3, 9809‐3, 9807‐3, 9872‐3, 9873‐3, 9874‐3, 9867‐3, 9891‐3, 9840‐3, 9910‐3, 9870‐3, 9931‐3, 9930‐3).

### 2.3 Statistical analysis

We used the Chi2/Fisher test to compare categorical variables and the Wilcoxon rank sum test for continuous variables according to patient accessibility to a specialised haematology unit. We then constructed a multivariate logistic regression model to determine the association between different covariables and access to a specialised haematology unit. For this modelling, we used a backward selection method to successively remove the variables whose significance was greater than 20%. We use Akaike Information Criterion (AIC) to choose the best fitted model. We systematically included the gender variable in the models even if it was not significant. For modelling purposes, we chose to exclude patients over 80 y‐o who died within the first 5 days after diagnostic and younger patients who died on the same day of diagnosis, assuming that these patients died due to their age or comorbidities before they had time to be referred to specialised haematology unit.

## 3 RESULTS

### 3.1 Patients characteristics according to their accessibility to specialised haematology unit

Of the 1039 incident AML cases, there were 529 men (51%) and 510 women (49%) with a median age of 73 years. There were 46% from Basse‐Normandie, 40% from Gironde and 14% from Côte d'Or (no statistical differences in AML subtypes were seen across diagnostic departments, result not shown). A total of 713 patients (69%) consulted in a SHU during their disease course and 326 patients (32%) did not (Table 1).

**TABLE 1:** Characteristics of patients according to their access to specialised haematology unit during their care pathway.

Characteristic	Haematological consultation			
	Overall, *N* = 1039^a^	Yes, *N* = 713^a^	No, *N* = 326^a^	*p*‐value^b^
Sex				0.095
Men	529 (51%)	376 (53%)	153 (47%)	
Women	510 (49%)	337 (47%)	173 (53%)	
Median age at diagnosis	73 (59, 82)	66 (53, 77)	83 (77, 88)	<0.001
Diagnostic department				0.8
Basse‐Normandie	478 (46%)	332 (47%)	146 (45%)	
Côte‐d'Or	147 (14%)	101 (14%)	46 (14%)	
Gironde	414 (40%)	280 (39%)	134 (41%)	
Type of hospital consulted				<0.001
Non‐academic hospital	274 (26%)	71 (10.0%)	203 (62%)	
Academic hospital	765 (74%)	642 (90%)	123 (38%)	
Medical entry unit				<0.001
Emergency	155 (15%)	96 (13%)	59 (18%)	
General medicine	650 (63%)	459 (64%)	191 (59%)	
Haematology unit	59 (5.7%)	59 (8.3%)	0 (0%)	
Specialised medical unit	154 (15%)	81 (11%)	73 (22%)	
Undetermined	21 (2.0%)	18 (2.5%)	3 (0.9%)	
EDI quintile				0.14
1	159 (16%)	111 (16%)	48 (15%)	
2	174 (17%)	131 (19%)	43 (13%)	
3	224 (22%)	155 (22%)	69 (21%)	
4	278 (27%)	187 (27%)	91 (28%)	
5	190 (19%)	120 (17%)	70 (22%)	
Unknown	14	9	5	
Charlson comorbidity index				<0.001
No comorbidities	489 (48%)	384 (55%)	105 (34%)	
Low‐mild comorbidities	360 (35%)	235 (33%)	125 (40%)	
Severe comorbidities	167 (16%)	85 (12%)	82 (26%)	
Unknown	23	9	14	
Karyotype/FISH				<0.001
Karyotype/FISH not done	196 (19%)	52 (7.3%)	144 (45%)	
Karyotype/FISH performed	832 (81%)	656 (93%)	176 (55%)	
Unknown	11	5	6	
AML subtype's				<0.001
AML‐RCA	63 (6.1%)	60 (8.4%)	3 (0.9%)	
PML‐RARA	48 (4.6%)	46 (6.5%)	2 (0.6%)	
AML‐MRC	121 (12%)	79 (11%)	42 (13%)	
Therapy‐related AML/MDS	251 (24%)	150 (21%)	101 (31%)	
AML‐NOS	129 (12%)	48 (6.7%)	81 (25%)	
AML others	427 (41%)	330 (46%)	97 (30%)	
AML secondary profile				0.005
de novo AML	765 (74%)	546 (77%)	219 (67%)	
t‐MDS	149 (14%)	87 (12%)	62 (19%)	
t‐AML	125 (12%)	80 (11%)	45 (14%)	
Initial cytogenetic prognostic staging				<0.001
Favourable	155 (15%)	145 (20%)	10 (3.1%)	
Intermediate	422 (41%)	319 (45%)	103 (32%)	
Adverse	228 (22%)	174 (24%)	54 (17%)	
Missing (Karyotype/FISH not done)	234 (23%)	75 (11%)	159 (49%)	
Initial treatment modalities				<0.001
Untreated patients	114 (11%)	35 (4.9%)	79 (25%)	
Non‐curative treatment	420 (41%)	194 (27%)	226 (70%)	
Curative treatment	499 (48%)	482 (68%)	17 (5.3%)	
Unknown	6	2	4	
Number of chemotherapy lines				<0.001
0	1 (1%)	0 (0%)	1 (0.8%)	
1 line	482 (63%)	368 (58%)	114 (88%)	
2 lines	186 (24%)	172 (27%)	14 (11%)	
>2lines	91 (12%)	90 (14%)	1 (0.8%)	
Unknown	279	83	196	
Cytological response to first line chemotherapy				<0.001
Failure	228 (34%)	155 (27%)	73 (77%)	
Partial response/Stable disease	96 (14%)	78 (14%)	18 (19%)	
Complete remission	345 (52%)	341 (59%)	4 (4.2%)	
Unknown	370	139	231	
Chemotherapy ± HSCT				<0.001
Chemotherapy +HSCT	184 (18%)	184 (26%)	0 (0%)	
Chemotherapy only	463 (45%)	399 (56%)	64 (20%)	
Untreated	386 (37%)	128 (18%)	258 (80%)	
Unknown	6	2	4	
Associated treatment				<0.001
No	448 (43%)	225 (32%)	223 (68%)	
Yes	591 (57%)	488 (68%)	103 (32%)	
Treated with immunotherapy				<0.001
No immunotherapy	950 (91%)	627 (88%)	323 (99%)	
Immunotherapy	89 (8.6%)	86 (12%)	3 (0.9%)	
Treated with radiotherapy				<0.001
No	1013 (97%)	687 (96%)	326 (100%)	
Yes	26 (2.5%)	26 (3.6%)	0 (0%)	
Inclusion in clinical trial				<0.001
No	570 (75%)	438 (70%)	132 (99%)	
Yes	193 (25%)	192 (30%)	1 (0.8%)	
Unknown	276	83	193	
MRD evaluation				<0.001
No	336 (32%)	319 (45%)	17 (5.2%)	
Yes	164 (16%)	164 (23%)	0 (0%)	
NA (untreated/Non‐curative treatment)	539 (52%)	230 (32%)	309 (95%)	
Vital status at 1 year				<0.001
Alive at 1 year	481 (46%)	421 (59%)	60 (18%)	
Died at 1 year	551 (53%)	290 (40.7%)	261 (80%)	
Lost to follow‐up at 1 year	7 (1%)	2 (0.3%)	5 (2%)	
Vital status at 5 years				<0.001
Alive at 5 years	139 (13%)	133 (19%)	6 (1.8%)	
Died at 5 years	760 (73%)	455 (64%)	305 (94%)	
Lost to follow‐up at 5 years	140 (14%)	125 (18%)	15 (4.6%)	

^a^ *n* (%); Median (IQR).

^b^ Fisher's Exact Test for Count Data; Wilcoxon rank sum test; Fisher's Exact Test for Count Data with simulated *p*‐value (based on 2000 replicates).

**FIGURE 1:**
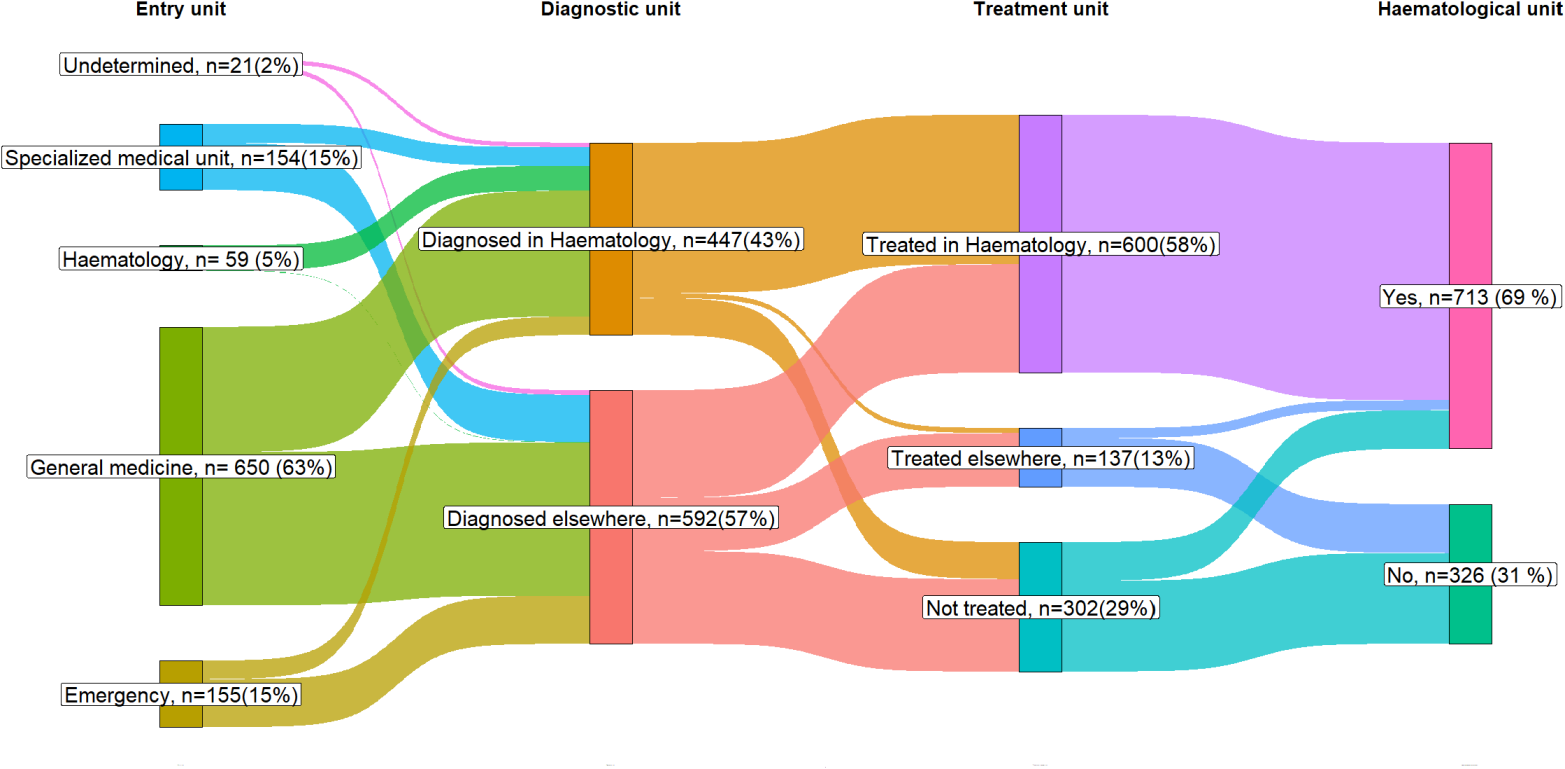
AML patient care pathways describing units for initial consultation, diagnosis and treatment.

**FIGURE 2:**
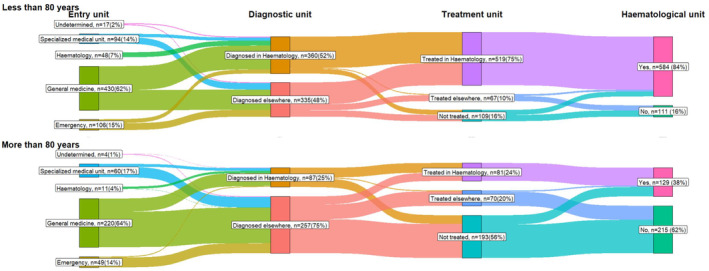
Patients care pathways according to first medical unit, diagnosis and treatment unit by age group.

Concerning the care pathway, the first medical contact was the general practitioner in 63% of cases (*n* = 650) with 71% (459/650) of access to a specialised haematology unit (the most frequently used care pathway). Similarly, 15% of patients started in an emergency unit (62% or 96/155 referred to the specialised haematology unit), 15% in a specialised medical unit (53% or 81/154 of referred to SHU) and 5% started directly in SHU (2% of missing data) (Table 1/Figure 1). An age difference was observed in the patients accessing a specialised haematology unit (Figure 2). During their care management, 86% of patients under 80 y‐o had access to SHU compared to 38% of older patients with either AML diagnosis or treatment decision (Figure 2). More specifically, AML was diagnosed by a trained haematologist in 52% of patients under 80 y‐o compared to 25% in those over 80 y‐o. Similarly, 74% of patients under 80 y‐o were treated in a SHU, compared to 24% of patients over 80 y‐o (Appendix S1 ‐ Table 3/Figure 2).

Patients who consulted in a SHU were younger (median age 66 vs. 83 y‐o), 90% of them went to an academic hospital (vs. 38% to a non‐academic hospital), but there was no statistical difference according to patient socio‐economic status (EDI quintile). Similarly, among patients who consulted in a SHU, 92% had access to cytogenetic testing (vs. 54% for those consulting outside a SHU); the proportion of AML‐MRC, t‐AML/MDS and AML‐NOS subtypes were less represented and 77% had de novo AML (vs. 67%). Patients admitted to SHU had more a favourable initial cytogenetic prognostic status (23% vs. 6%), less comorbidities (54% with no comorbidity vs. 32%) and more frequently received curative treatment 68% (vs. 5%). Additionally, 14 (11%) of the over‐80 y‐o patients who consulted a trained haematologist received curative treatment (vs. <1% over 80 y‐o who did not see a trained haematologist) (see details in the Appendix S1 ‐ Table 3). Among patients who consulted in a SHU, 58% (*n* = 368) received one line of chemotherapy (vs. 88%, *n* = 114 of non‐SHU patients), 27% (*n* = 172) received two lines of chemotherapy (vs. 11%, *n* = 14 of non‐SHU patients) and 12% (*n* = 91) received more than two lines of chemotherapy (vs. 0.8%, *n* = 1 of non‐SHU patients). Among patients who received curative treatment, the first‐line complete remission rate was 59% for patients who consulted in a SHU (vs. 4.2%, *p* = 0.001). Patients admitted to a SHU had greater access to associated treatment related to chemotherapy 68% (vs. 32%, *n* = 103). Access to haematopoietic stem cell transplantation (HSCT) and minimal residual disease (MRD) was reserved strictly for patients treated in SHU. Similarly, immunotherapy, radiotherapy and inclusion in clinical trials were almost exclusively seen among patients who had consulted a trained haematologist (Table 1).

### 3.2 Factors associated with access to specialised haematology units

In the univariate model, factors limiting access to the SHU were being in the age group above 50 years old, emergency referral (OR, 0.77; 95% CI, 0.58–1.01), specialised medical referral (OR, 0.11, 95% CI, 0.08–0.15), patients with low‐mild (OR, 0.52; 95% CI, 0.38–0.71) or severe (OR, 0.27, 95% CI, 0.19–0.40) comorbidities. Similarly, other factors such as being diagnosed with AML‐MRC (OR, 0.09; 95% CI, 0.02–0.27), t‐AML (OR, 0.08, 95% CI, 0.02–0.21), AML‐NOS (OR, 0.04, 95% CI, 0.01–0.11), AML‐others (OR, 0.18; CI, 0.04–0.50) or an intermediate (OR, 0.20; 95% CI, 0.09–0.39), adverse cytogenetic prognosis (OR, 0.20; 95% CI, 0.09–0.41) were also factors limiting access to SHU. In addition, based on EDI quintiles, patients with lower socio‐economic status had less access to SHUs compared to the higher income group (Table 2).

**TABLE 2:** Regression models of factors influencing access to specialised haematology unit.

Characteristic	Descriptive analysis			Univariate model			Multivariate model AIC = 664.3		
	Overall, *N* = 1010^a^	No, *N* = 298^a^	Yes, *N* = 712^a^	OR^b^	95% CI^b^	*p*‐value	OR^b^	95% CI^b^	*p*‐value
Sex						0.11			>0.9
Men	517 (100%)	141 (27%)	376 (73%)	—	—		—	—	
Women	493 (100%)	157 (32%)	336 (68%)	0.80	0.61, 1.05		1.00	0.66, 1.51	>0.9
Age in class						<0.001			<0.001
[0,50)	147 (100%)	4 (2.7%)	143 (97%)	—	—		—	—	
[50,65)	199 (100%)	17 (8.5%)	182 (91%)	0.30	0.08, 0.83		0.65	0.17, 2.03	0.5
[65,80)	347 (100%)	89 (26%)	258 (74%)	0.08	0.02, 0.20		0.41	0.11, 1.15	0.12
[80,101]	317 (100%)	188 (59%)	129 (41%)	0.02	0.01, 0.05		0.14	0.04, 0.38	<0.001
Diagnostic department						0.96			
Basse‐Normandie	467 (100%)	136 (29%)	331 (71%)	—	—		—	—	
Côte‐d'Or	143 (100%)	42 (29%)	101 (71%)	0.99	0.66, 1.50				
Gironde	400 (100%)	120 (30%)	280 (70%)	0.96	0.72, 1.29				
EDI quintile						0.15			
1	155 (100%)	44 (28%)	111 (72%)	—	—		—	—	
2	169 (100%)	39 (23%)	130 (77%)	1.32	0.80, 2.18				
3	218 (100%)	63 (29%)	155 (71%)	0.98	0.62, 1.54				
4	268 (100%)	81 (30%)	187 (70%)	0.92	0.59, 1.41				
5	186 (100%)	66 (35%)	120 (65%)	0.72	0.45, 1.14				
Unknown	14	5	9						
Treatment facility type						<0.001			<0.001
Non‐academic hospital	261 (100%)	190 (73%)	71 (27%)	—	—		—	—	
Academic hospital	749 (100%)	108 (14%)	641 (86%)	15.9	11.4, 22.5		8.87	5.64, 14.2	<0.001
Emergency consultation						0.058			<0.001
No	605 (100%)	165 (27%)	440 (73%)	—	—		—	—	
Yes	405 (100%)	133 (33%)	272 (67%)	0.77	0.58, 1.01		0.28	0.18, 0.44	<0.001
Specialised unit consultation						<0.001			<0.001
No	565 (100%)	63 (11%)	502 (89%)	—	—		—	—	
Yes	445 (100%)	235 (53%)	210 (47%)	0.11	0.08, 0.15		0.12	0.07, 0.18	<0.001
General medicine consultation						0.065			0.4
No	363 (100%)	120 (33%)	243 (67%)	—	—		—	—	
Yes	647 (100%)	178 (28%)	469 (72%)	1.30	0.98, 1.72		0.82	0.53, 1.26	0.4
Charlson comorbidity index						<0.001			0.005
No comorbidities	479 (100%)	96 (20%)	383 (80%)	—	—		—	—	
Low‐mild comorbidities	349 (100%)	114 (33%)	235 (67%)	0.52	0.38, 0.71		0.80	0.50, 1.28	0.3
Severe comorbidities	163 (100%)	78 (48%)	85 (52%)	0.27	0.19, 0.40		0.39	0.21, 0.69	0.001
Unknown	19	10	9						
AML sub‐type						<0.001			0.074
AML‐RCA	63 (100%)	3 (4.8%)	60 (95%)	—	—		—	—	
PML‐RARA	48 (100%)	2 (4.2%)	46 (96%)	1.15	0.18, 9.00		0.42	0.04, 5.06	0.5
AML‐MRC	121 (100%)	42 (35%)	79 (65%)	0.09	0.02, 0.27		0.19	0.03, 0.97	0.065
Therapy‐related AML/MDS	249 (100%)	99 (40%)	150 (60%)	0.08	0.02, 0.21		0.13	0.02, 0.62	0.020
AML‐NOS	108 (100%)	61 (56%)	47 (44%)	0.04	0.01, 0.11		0.10	0.01, 0.51	0.011
AML others	421 (100%)	91 (22%)	330 (78%)	0.18	0.04, 0.50		0.15	0.02, 0.70	0.028
Cytogenetic initial prognosis						<0.001			0.007
Favourable	154 (100%)	9 (5.8%)	145 (94%)	—	—		—	—	
Intermediate	418 (100%)	99 (24%)	319 (76%)	0.20	0.09, 0.39		0.92	0.32, 2.35	0.9
Adverse	227 (100%)	53 (23%)	174 (77%)	0.20	0.09, 0.41		0.80	0.27, 2.13	0.7
Missing (Karyotype/FISH not done)	211 (100%)	137 (65%)	74 (35%)	0.03	0.02, 0.07		0.37	0.12, 1.01	0.062

*Note*: AIC = 664.

^a^ *n* (%).

^b^ OR = Odds Ratio; CI = Confidence Interval.

After adjustment, factors limiting access to a SHU were aged over 80 years old (ORa, 0.14; 95% CI, 0.04–0.38), emergency referral (ORa, 0.28; 95% CI, 0.18–0.44), or specialised unit referral (ORa, 0.12; 95% CI, 0.07–0.18). Also, patients with severe comorbidities (ORa, 0.39; 95% CI, 0.21–0.69) and patients with subtypes t‐AML/MDS (ORa, 0.13; 95% CI, 0.02–0.62), AML‐NOS (ORa, 0.10; 95% CI, 0.01–0.51) or AML‐others (ORa, 0.15; 95% CI, 0.02–0.70) were less likely to be sent to a SHU. However, being admitted to an academic hospital increased referral to SHU consultation by 8.87 times (Table 2).

## 4 DISCUSSION

Our population‐based study has investigated the impact of non‐biological factors on AML patient care pathways including those that could directly and/or indirectly influence treatment management. An added strength of our study is that, the former analysis was performed alongside an assessment of the impact of known prognostic parameters, including AML subtype and cytogenetic risk group. By using this combined approach, we were able to demonstrate the importance of consulting in a specialised haematology unit during the care pathway. This seems to have an impact on access to the best diagnostic tools and curative treatments, which in turn are well described in the literature as factors improving the overall survival of AML patients.^13^, ^22^

Several studies have investigated the impact of treatment facility type upon survival in AML, without evaluating the impact of access to specialised haematology units.^16^, ^18^ The present work shows that this should be taken into consideration since patients who are managed in academic hospitals have 8.87 times more access to specialised haematology unit (Figure 1). Access to a specialised haematology unit does not seem to be related to patient socio‐economic status but rather to biological or clinical factors and potentially, to the accessibility of specialised AML treatment facilities in the patient geographical area of residence. However, a trend for the most deprived patients to have less access to a specialised haematology unit was observed in the univariate analysis, although this was not confirmed in the multivariate model. In the absence of individual measures of deprivation, the ecological measure (EDI quintile) reflects both the contextual and individual deprivation of the patient, and as such, does not fully represent the patient's socio‐economic status.

During the period up to formal diagnosis of AML, patients may consult several clinical units and undergo various additional examinations, leading to rather diverse care pathways. Several factors, including clinical symptoms, age, patient geographical location, and other socio‐economic factors influence this.^15^ Our data show that advanced age remains a limitation for access to the specialised haematology unit, as observed in patients with the AML‐NOS subtype (median age = 84 vs. 73 years on average; 37% access to the SHU vs. 69% on average; OR = 0.10, 95% CI, 0.01–0.51). Lack of referral of these older patients to a specialised haematology unit resulted in less access to cytogenetic analysis (39% vs. 80% on average) thus potentially explaining their low access to curative treatment (18% vs. 48% on average per subtype, result not shown). Overall, this may negatively impact survival in this patient group. This is problematic because the incidence of AML continues to increase in this age group since 1990.^1^

More generally, our work highlights the impact of the AML care pathway on access to cytogenetic testing, an essential examination for accurate AML diagnosis and prognostic classification, according to ELN guidelines. Indeed, 45% of patients not referred to a specialised haematology unit did not receive cytogenetic testing (vs. 7.3% among SHU patients). Furthermore, of those AML patients who did not have access to cytogenetic testing, 91% were diagnosed with poor prognosis AML subtypes (*n* = 196) (57% AML‐NOS, 22% T‐AML, and 12% Other‐AML). It is probable that cytogenetics would allowed re‐classification of at least some of these cases to other AML subtypes. For these patients, it is possible that the lack of transfer to a specialised haematology unit, the limitation in diagnostic investigations, and / or the lack of intensive therapy derives from a perceived limited benefit of these strategies on quality of life and vital prognosis. However, a possible treatment could be claimed, if the investigations had been completed.

The same reasoning can be applied to the patients with severe comorbidities and who were potentially monitored elsewhere for a previous pathology. Indeed, severe comorbidities when combined with adverse cytogenetics in some AML subtypes can negatively impact patient access to a specialised haematology unit, for the presumed limited benefit this might bring.^23^, ^24^, ^25^, ^26^

Quite strikingly, we found that 74% (203/274) of AML patients who consulted at non‐academic hospitals, were subsequently managed in a non‐haematology unit. This may simply reflect the absence of SHU in non‐academic hospitals. Similarly, it is possible that these patients died before they could be transferred to a hospital with a specialised haematology unit (death represents a competitive event for access to SHU, for which we have minimised the impact in the logistic modelling). By contrast, admission to an academic‐hospital would favour access to a specialised haematology unit (ORa = 8.87), and thus optimal AML diagnosis and prognostic stratification with consequent increased probability of receiving curative treatment. Such treatment decisions by expert haematologists are further supported by access to expert facilities for management of adverse events in academic centres.^17^ It should be noted that specialised haematology unit, tend to admit the better prognosis AML patients.^16^

More importantly, haematopoietic stem cell transplantation, immunotherapy, radiotherapy, MRD evaluation and access to clinical trials were strictly reserved for patients who were seen by a trained haematologist. Given the positive impact of transplantation on the survival of AML patients,^27^ and the innovative therapies proposed in clinical trials,^28^, ^29^ working to improve patient access to specialised haematology unit will be essential to improve AML patient survival in the general population.

Finally, based on patient clinical characteristics, we split patients into eligible (age ≤75 years without severe comorbidities) and non‐eligible for treatment (over 75 years with sever comorbidities) among patients alive 5 days after diagnosis. Regarding the age boundary, we followed the age‐related Ferrara unfitness criterion.^33^ By this method, we could show that 77% of non‐eligible patients receive treatment (28% and 49% for curative and palliative care respectively) when they visit a specialised haematology unit versus 42% (2.8% and 39% for curative and palliative care respectively) when they did not (*p* < 0.001) (Appendix S1 ‐ Table 4). These results show the importance of a trained haematologist for unfit AML patients. Indeed, with the advent of oral chemotherapy agents facilitating outpatient care, and non‐intensive chemotherapies (e.g. azacytidine venetoclax combination),^30^, ^31^ it can be assumed that the trained haematologist attempts to use these new therapeutic tools to manage unfit patients. The fact that the seven patients over 80 years old who were enrolled in a clinical trial were recruited by trained haematologist tends to support this notion (Table 1). By contrast, unfit patients seen elsewhere do not have access to these new therapies, especially as an increasing number of studies suggest they should be treated with non‐intensive chemotherapies.^29^, ^32^, ^33^

Our study does present a number of limitations which need to be addressed. First, we categorised the EDI based on quintiles and such class variables are potentially less informative.^34^ The EDI‐quintile may, however, reflect the level of access to adequate health care facilities, as determined by the geographical area of the patient's residence. Our results also showed that the presence of severe co‐morbidities can limit patient access to specialised haematology units. However, a higher prevalence of severe co‐morbidities is seen among the most deprived patients, as defined by EDI.^24^ To uncover how the socio‐economic status affects access to specialised care facilities and the role of co‐morbidities for AML patients, information on distance and travel times to specialised care facilities, individual comorbidities, would be required. These data were not available in our study as is the case in other reports of similar design.^19^, ^35^

A second limitation concerns our finding that consultation in non‐haematological medical units is negatively correlated (ORa = 0.12, 95% CI, 0.07–0.18) with access to specialised haematology unit. We hypothesised that this reflects more complex clinical situations that require transfer to non‐haematological units, despite a diagnosis of AML. Again, in the absence of detailed information on the clinical signs justifying the lack of consultation in a specialised haematology unit, we cannot rule out the hypothesis that these patients were advised by a specialised haematologist (e.g. during a multidisciplinary consultation meeting) or that they wished not to be treated. Such information was not available in our study.

These limitations however do not affect our main conclusions, and our findings raise the question of what therapeutic approach would have been taken if these patients had consulted in a specialised haematology unit during their course of care. To this end, in the next stage of our project, we will apply causal mediation techniques to quantify how accessing a specialised haematology unit causally contributes to the likelihood of receiving a curative treatment and impacts differential AML patient net survival.

## 5 CONCLUSION

In this study, we show for the first time that well‐known clinical and biological prognostic factors limit the access of AML patients to a specialised haematology unit, which in turns strongly impedes access to cytogenetic analyses and curative treatments. Our study highlights the importance of a haematological unit referral, or a consultation in an academic hospital, for AML patients to have the best chance of being optimally treated according to individual disease risk factors and comorbidities.

## AUTHOR CONTRIBUTIONS

**Kueshivi Midodji ATSOU:** Data curation (lead); formal analysis (lead); methodology (lead); writing – original draft (lead). **Bernard Rachet:** Formal analysis (supporting); methodology (supporting); supervision (supporting); validation (supporting); writing – review and editing (lead). **Edouard Cornet:** Data curation (supporting); writing – review and editing (equal). **Marie‐Lorraine Chretien:** Conceptualization (equal); resources (equal); writing – review and editing (equal). **Cédric Rossi:** Conceptualization (equal); resources (equal); writing – review and editing (equal). **Laurent Remontet:** Conceptualization (supporting); methodology (supporting); writing – review and editing (equal). **Laurent Roche:** Conceptualization (supporting); methodology (supporting); writing – review and editing (equal). **Roch Giorgi:** Conceptualization (supporting); formal analysis (supporting); methodology (supporting); writing – review and editing (supporting). **Sophie Gauthier:** Conceptualization (equal); data curation (equal). **Stéphanie Girard:** Conceptualization (equal); data curation (equal). **Johann Bôckle:** Conceptualization (equal); data curation (equal). **Stéphane Kroudia Wasse:** Resources (equal); writing – review and editing (equal). **Hélène Rachou:** Data curation (equal). **Laïla Bouzid:** Data curation (equal). **Jean‐Marc Poncet:** Data curation (equal). **Sébastien Orazio:** Methodology (supporting). **Alain Monnereau:** Resources (equal); supervision (supporting); writing – review and editing (supporting). **Xavier Troussard:** Resources (equal); supervision (supporting); writing – review and editing (supporting). **Morgane Mounier:** Conceptualization (lead); funding acquisition (lead); investigation (lead); methodology (supporting); project administration (lead); resources (lead); writing – review and editing (supporting). **Marc Maynadié:** Conceptualization (equal); funding acquisition (lead); investigation (supporting); project administration (supporting); resources (supporting); supervision (supporting); writing – original draft (supporting); writing – review and editing (supporting).

## FUNDING INFORMATION

This study was supported by research funding from Fonds Européen de développement regional (FEDER: programme opérationnel FEDER‐FSE Bourgogne 2014–2020) and from Institut National du Cancer (Projet INCa‐SHS‐ESP, n°2018–124).

## CONFLICT OF INTEREST

The authors declare no competing financial interests.

## ETHICAL APPROVAL STATEMENT

This study was authorised by the CNIL (Commission Nationale Informatique & Libertés) and received a favourable opinion from the ethics committee of the CESRESS (Comité d'Éthique et Scientifique pour les Recherches, les études et Évaluations dans le domaine de Santé) under the reference number MLD/CBO/AR2111097.

## Supporting information


Appendix S1

## Data Availability

The data sets reported in this study are available on reasonable request from the corresponding author.
