# Supplementary material for: Factors influencing access to specialised haematology units during acute myeloblastic leukaemia patient care: A population‐based study in France
Source: Cancer Med. 2023 Jan 29;12(7):8911–23. doi: 10.1002/cam4.5645 (PMC10134294; doi:10.1002/cam4.5645)
Supplement: Supplementary file 1 — Appendix S1 [file CAM4-12-8911-s001.docx]

APPENDIX

| **Table 3*:*** Characteristics of patients according to group age and their access to specialised haematology unit | | | | | | | | |  |
| --- | --- | --- | --- | --- | --- | --- | --- | --- | --- |
|  | **Less than 80 years** | | | | **More than 80 years** | | | |  |
| **Characteristic** | **Overall,**  **N = 695^1^** | **No,**  **N = 111^1^** | **Yes,**  **N = 584^1^** | **p-value^2^** | **Overall,**  **N = 344^1^** | **No,**  **N = 215^1^** | **Yes,**  **N = 129^1^** | **p-value^2^** | |
| **Sex** |  |  |  | 0.4 |  |  |  | 0.4 | |
| Men | 384 (55%) | 66 (59%) | 318 (54%) |  | 145 (42%) | 87 (40%) | 58 (45%) |  | |
| Women | 311 (45%) | 45 (41%) | 266 (46%) |  | 199 (58%) | 128 (60%) | 71 (55%) |  | |
| **Median age** | 64 (52, 73) | 74 (68, 77) | 62 (50, 71) | <0.001 | 85.0 (82.0, 88.0) | 86.0 (83.0, 89.0) | 83.0 (81.0, 86.0) | <0.001 | |
| **Diagnostic department** |  |  |  | 0.9 |  |  |  | 0.086 | |
| Basse-Normandie | 312 (45%) | 52 (47%) | 260 (45%) |  | 166 (48%) | 94 (44%) | 72 (56%) |  | |
| Dijon | 98 (14%) | 14 (13%) | 84 (14%) |  | 49 (14%) | 32 (15%) | 17 (13%) |  | |
| Gironde | 285 (41%) | 45 (41%) | 240 (41%) |  | 129 (38%) | 89 (41%) | 40 (31%) |  | |
| **Treatment facility type** |  |  |  | <0.001 |  |  |  | <0.001 | |
| Non-academic hospital | 103 (15%) | 71 (64%) | 32 (5.5%) |  | 171 (50%) | 132 (61%) | 39 (30%) |  | |
| Academic hospital | 592 (85%) | 40 (36%) | 552 (95%) |  | 173 (50%) | 83 (39%) | 90 (70%) |  | |
| **Medical entry unit** |  |  |  | <0.001 |  |  |  | <0.001 | |
| Emergency | 106 (15%) | 19 (17%) | 87 (15%) |  | 49 (14%) | 40 (19%) | 9 (7.0%) |  | |
| General medicine | 430 (62%) | 56 (50%) | 374 (64%) |  | 220 (64%) | 135 (63%) | 85 (66%) |  | |
| Haematology | 48 (6.9%) | 0 (0%) | 48 (8.2%) |  | 11 (3.2%) | 0 (0%) | 11 (8.5%) |  | |
| Specialized medical unit | 94 (14%) | 35 (32%) | 59 (10%) |  | 60 (17%) | 38 (18%) | 22 (17%) |  | |
| Undetermined | 17 (2.4%) | 1 (0.9%) | 16 (2.7%) |  | 4 (1.2%) | 2 (0.9%) | 2 (1.6%) |  | |
| **EDI quintile** |  |  |  | 0.2 |  |  |  | 0.9 | |
| 1 | 113 (17%) | 20 (19%) | 93 (16%) |  | 46 (13%) | 28 (13%) | 18 (14%) |  | |
| 2 | 124 (18%) | 13 (12%) | 111 (19%) |  | 50 (15%) | 30 (14%) | 20 (16%) |  | |
| 3 | 156 (23%) | 29 (27%) | 127 (22%) |  | 68 (20%) | 40 (19%) | 28 (22%) |  | |
| 4 | 171 (25%) | 23 (21%) | 148 (26%) |  | 107 (31%) | 68 (32%) | 39 (30%) |  | |
| 5 | 120 (18%) | 23 (21%) | 97 (17%) |  | 70 (21%) | 47 (22%) | 23 (18%) |  | |
| Unknown | 11 | 3 | 8 |  | 3 | 2 | 1 |  | |
| **Charlson comorbidity index** |  |  |  | <0.001 |  |  |  | 0.8 | |
| No comorbidities | 365 (53%) | 31 (28%) | 334 (58%) |  | 124 (38%) | 74 (37%) | 50 (40%) |  | |
| Low-mild comorbidities | 228 (33%) | 42 (38%) | 186 (32%) |  | 132 (40%) | 83 (41%) | 49 (40%) |  | |
| Severe comorbidities | 97 (14%) | 37 (34%) | 60 (10%) |  | 70 (21%) | 45 (22%) | 25 (20%) |  | |
| Unknown | 5 | 1 | 4 |  | 18 | 13 | 5 |  | |
| **Karyotype/FISH** |  |  |  | <0.001 |  |  |  | <0.001 | |
| Karyotype and fish not done | 51 (7.4%) | 28 (26%) | 23 (4.0%) |  | 145 (43%) | 116 (55%) | 29 (23%) |  | |
| Karyotype or fish realized | 638 (93%) | 81 (74%) | 557 (96%) |  | 194 (57%) | 95 (45%) | 99 (77%) |  | |
| Unknown | 6 | 2 | 4 |  | 5 | 4 | 1 |  | |
|  |  |  |  |  |  |  |  |  | |
| **AML sub-type** |  |  |  | <0.001 |  |  |  | 0.001 | |
| AML-RCA | 56 (8.1%) | 1 (0.9%) | 55 (9.4%) |  | 7 (2.0%) | 2 (0.9%) | 5 (3.9%) |  | |
| PML-RARA | 43 (6.2%) | 1 (0.9%) | 42 (7.2%) |  | 5 (1.5%) | 1 (0.5%) | 4 (3.1%) |  | |
| AML-MRC | 66 (9.5%) | 8 (7.2%) | 58 (9.9%) |  | 55 (16%) | 34 (16%) | 21 (16%) |  | |
| Therapy related AML/MDS | 157 (23%) | 53 (48%) | 104 (18%) |  | 94 (27%) | 48 (22%) | 46 (36%) |  | |
| AML-NOS | 45 (6.5%) | 15 (14%) | 30 (5.1%) |  | 84 (24%) | 66 (31%) | 18 (14%) |  | |
| AML others | 328 (47%) | 33 (30%) | 295 (51%) |  | 99 (29%) | 64 (30%) | 35 (27%) |  | |
| **AML secondary profile** |  |  |  | <0.001 |  |  |  | 0.011 | |
| de novo AML | 519 (75%) | 53 (48%) | 466 (80%) |  | 246 (72%) | 166 (77%) | 80 (62%) |  | |
| t-MDS | 92 (13%) | 35 (32%) | 57 (9.8%) |  | 57 (17%) | 27 (13%) | 30 (23%) |  | |
| t-AML | 84 (12%) | 23 (21%) | 61 (10%) |  | 41 (12%) | 22 (10%) | 19 (15%) |  | |
| **Initial cytogenetic prognostic staging** | |  |  | <0.001 |  |  |  | 0.15 | |
| Favourable | 141 (23%) | 5 (6.5%) | 136 (25%) |  | 14 (7.6%) | 5 (5.6%) | 9 (9.6%) |  | |
| Intermediate | 302 (49%) | 48 (62%) | 254 (47%) |  | 120 (65%) | 55 (61%) | 65 (69%) |  | |
| Adverse | 178 (29%) | 24 (31%) | 154 (28%) |  | 50 (27%) | 30 (33%) | 20 (21%) |  | |
| Unknown | 74 | 34 | 40 |  | 160 | 125 | 35 |  | |
|  |  |  |  |  |  |  |  |  | |
| **Initial treatment modalities** |  |  |  | <0.001 |  |  |  | <0.001 | |
| Untreated patients | 53 (7.7%) | 27 (25%) | 26 (4.5%) |  | 61 (18%) | 52 (25%) | 9 (7.0%) |  | |
| Non-curative treatment | 156 (23%) | 68 (62%) | 88 (15%) |  | 264 (77%) | 158 (75%) | 106 (82%) |  | |
| Curative treatment | 483 (70%) | 15 (14%) | 468 (80%) |  | 16 (4.7%) | 2 (0.9%) | 14 (11%) |  | |
| Unknown | 3 | 1 | 2 |  | 3 | 3 | 0 |  | |
| **Number of chemotherapy lines** |  |  |  | <0.001 |  |  |  | 0.5 | |
| 0 | 0 (0%) | 0 (0%) | 0 (0%) |  | 1 (0.6%) | 1 (1.3%) | 0 (0%) |  | |
| 1 line | 337 (57%) | 46 (85%) | 291 (54%) |  | 145 (87%) | 68 (89%) | 77 (85%) |  | |
| 2 lines | 169 (28%) | 8 (15%) | 161 (30%) |  | 17 (10%) | 6 (7.9%) | 11 (12%) |  | |
| >2lines | 87 (15%) | 0 (0%) | 87 (16%) |  | 4 (2.4%) | 1 (1.3%) | 3 (3.3%) |  | |
| Unknown | 102 | 57 | 45 |  | 177 | 139 | 38 |  | |
| **Cytological response to first line chemotherapy** |  |  |  | <0.001 |  |  |  | 0.033 | |
| Failure | 137 (26%) | 27 (75%) | 110 (22%) |  | 91 (67%) | 46 (78%) | 45 (59%) |  | |
| Partial response/Stable disease | 61 (11%) | 6 (17%) | 55 (11%) |  | 35 (26%) | 12 (20%) | 23 (30%) |  | |
| Complete remission | 336 (63%) | 3 (8.3%) | 333 (67%) |  | 9 (6.7%) | 1 (1.7%) | 8 (11%) |  | |
| Unknown | 161 | 75 | 86 |  | 209 | 156 | 53 |  | |
|  |  |  |  |  |  |  |  |  | |
| **Chemotherapy ± HSCT** |  |  |  | <0.001 |  |  |  | <0.001 | |
| Chemotherapy+ HSCT | 184 (27%) | 0 (0%) | 184 (32%) |  | 0 (0%) | 0 (0%) | 0 (0%) |  | |
| Chemotherapy only | 367 (53%) | 34 (31%) | 333 (57%) |  | 96 (28%) | 30 (14%) | 66 (51%) |  | |
| Untreated | 141 (20%) | 76 (69%) | 65 (11%) |  | 245 (72%) | 182 (86%) | 63 (49%) |  | |
| Unknown | 3 | 1 | 2 |  | 3 | 3 | 0 |  | |
| **Associated treatment** |  |  |  | <0.001 |  |  |  | 0.018 | |
| No | 220 (32%) | 70 (63%) | 150 (26%) |  | 228 (66%) | 153 (71%) | 75 (58%) |  | |
| Yes | 475 (68%) | 41 (37%) | 434 (74%) |  | 116 (34%) | 62 (29%) | 54 (42%) |  | |
| **Treated with immunotherapy** |  |  |  | <0.001 |  |  |  | >0.9 | |
| Immunotherapy | 88 (13%) | 2 (1.8%) | 86 (15%) |  | 1 (0.3%) | 1 (0.5%) | 0 (0%) |  | |
| No immunotherapy | 607 (87%) | 109 (98%) | 498 (85%) |  | 343 (100%) | 214 (100%) | 129 (100%) |  | |
| **Treated with radiotherapy** |  |  |  | 0.014 |  |  |  | >0.9 | |
| No | 669 (96%) | 111 (100%) | 558 (96%) |  | 344 (100%) | 215 (100%) | 129 (100%) |  | |
| Yes | 26 (3.7%) | 0 (0%) | 26 (4.5%) |  | 0 (0%) | 0 (0%) | 0 (0%) |  | |
|  |  |  |  |  |  |  |  |  | |
|  |  |  |  |  |  |  |  |  | |
|  |  |  |  |  |  |  |  |  | |
| **Inclusion in clinical trial** |  |  |  | <0.001 |  |  |  | 0.016 | |
| No | 408 (69%) | 54 (98%) | 354 (66%) |  | 162 (96%) | 78 (100%) | 84 (92%) |  | |
| Yes | 186 (31%) | 1 (1.8%) | 185 (34%) |  | 7 (4.1%) | 0 (0%) | 7 (7.7%) |  | |
| Unknown | 101 | 56 | 45 |  | 175 | 137 | 38 |  | |
| ^1^n (%); Median (IQR) | | | | | | | | |  |
| ^2^Fisher's Exact Test for Count Data; Wilcoxon rank sum test; Fisher's Exact Test for Count Data with simulated p-value (based on 2000 replicates) | | | | | | | | |  |
|  | | | | | | | | |  |

| **Table 4:** Eligibility to treatment, access to SHU and treatment received among patients alive 5 days after their diagnostic | | | | | | | | |
| --- | --- | --- | --- | --- | --- | --- | --- | --- |
|  | **Eligible** | | | | **Non-eligible** | | | |
| **Characteristic** | **Overall, N = 486^1^** | **Non-access to SHU, N = 35^1^** | **Access to SHU, N = 451^1^** | **p-value^2^** | **Overall, N = 495^1^** | **Non-access to SHU, N = 246^1^** | **Access to SHU, N = 249^1^** | **p-value^2^** |
| **Treatment modalities** |  |  |  | <0.001 |  |  |  | <0.001 |
| No treatment received | 39 (8.0%) | 18 (51%) | 21 (4.7%) |  | 200 (40%) | 143 (58%) | 57 (23%) |  |
| Treatment received | 447 (92%) | 17 (49%) | 430 (95%) |  | 295 (60%) | 103 (42%) | 192 (77%) |  |
| **Specific treatment received** |  |  |  | <0.001 |  |  |  | <0.001 |
| No treatment | 23 (4.7%) | 10 (29%) | 13 (2.9%) |  | 64 (13%) | 43 (17%) | 21 (8.4%) |  |
| Supportive care | 16 (3.3%) | 8 (23%) | 8 (1.8%) |  | 136 (27%) | 100 (41%) | 36 (14%) |  |
| Palliative care | 32 (6.6%) | 10 (29%) | 22 (4.9%) |  | 218 (44%) | 96 (39%) | 122 (49%) |  |
| Curative treatment | 415 (85%) | 7 (20%) | 408 (90%) |  | 77 (16%) | 7 (2.8%) | 70 (28%) |  |
| ^1^n (%) | | | | | | | | |
| ^2^Fisher's Exact Test for Count Data; Fisher's Exact Test for Count Data with simulated p-value  (based on 2000 replicates) | | | | | | | | |

| **Table 5:** Characteristics of patients according to their care pathway | | | | | | | | | | | | | | | | | | | | |
| --- | --- | --- | --- | --- | --- | --- | --- | --- | --- | --- | --- | --- | --- | --- | --- | --- | --- | --- | --- | --- |
| **Characteristic** | **Overall,**  **N = 1,039^1^** | **Emergency only/other,**  **N = 59^1^** | **Emergency🡺 SHU, N = 99^1^** | | **General Medicine only/other,**  **N = 191^1^** | | **General Medicine🡺SHU,**  **N = 457^1^** | | **Haematology, N = 59^1^** | | **SHU,**  **N = 72^1^** | | **Specialized medical unit🡺 SHU,**  **N = 78^1^** | | **Missing step in care pathway,**  **N = 24^1^** | | **p-value^2^** | |  |  |
| **Haematological consultation** |  |  |  | |  | |  | |  | |  | |  | |  | | <0.001 | |  |  |
| No | 326 (31%) | 59 (100%) | 0 (0%) | | 191 (100%) | | 0 (0%) | | 0 (0%) | | 72 (100%) | | 0 (0%) | | 4 (17%) | |  | |  |  |
| Yes | 713 (69%) | 0 (0%) | 99 (100%) | | 0 (0%) | | 457 (100%) | | 59 (100%) | | 0 (0%) | | 78 (100%) | | 20 (83%) | |  | |  |  |
| **Sex** |  |  |  | |  | |  | |  | |  | |  | |  | | 0.070 | |  |  |
| Men | 529 (51%) | 25 (42%) | 52 (53%) | | 84 (44%) | | 232 (51%) | | 38 (64%) | | 42 (58%) | | 45 (58%) | | 11 (46%) | |  | |  |  |
| Women | 510 (49%) | 34 (58%) | 47 (47%) | | 107 (56%) | | 225 (49%) | | 21 (36%) | | 30 (42%) | | 33 (42%) | | 13 (54%) | |  | |  |  |
| **Age group** |  |  |  | |  | |  | |  | |  | |  | |  | | <0.001 | |  |  |
| Less than 80 years | 695 (67%) | 20 (34%) | 90 (91%) | | 55 (29%) | | 372 (81%) | | 48 (81%) | | 34 (47%) | | 56 (72%) | | 20 (83%) | |  | |  |  |
| More than 80 years | 344 (33%) | 39 (66%) | 9 (9.1%) | | 136 (71%) | | 85 (19%) | | 11 (19%) | | 38 (53%) | | 22 (28%) | | 4 (17%) | |  | |  |  |
| **Median age** | 73 (59, 82) | 84 (77, 88) | 61 (39, 71) | | 84 (78, 88) | | 66 (53, 77) | | 68 (62, 78) | | 80 (74, 87) | | 70 (58, 81) | | 70 (62, 78) | | <0.001 | |  |  |
| **Diagnostic department** |  |  |  | |  | |  | |  | |  | |  | |  | | <0.001 | |  |  |
| Basse-Normandie | 478 (46%) | 21 (36%) | 42 (42%) | | 85 (45%) | | 211 (46%) | | 27 (46%) | | 37 (51%) | | 39 (50%) | | 16 (67%) | |  | |  |  |
| Dijon | 147 (14%) | 9 (15%) | 15 (15%) | | 25 (13%) | | 51 (11%) | | 17 (29%) | | 11 (15%) | | 11 (14%) | | 8 (33%) | |  | |  |  |
| Gironde | 414 (40%) | 29 (49%) | 42 (42%) | | 81 (42%) | | 195 (43%) | | 15 (25%) | | 24 (33%) | | 28 (36%) | | 0 (0%) | |  | |  |  |
| **Medical entry unit** |  |  |  | |  | |  | |  | |  | |  | |  | | <0.001 | |  |  |
| Emergency | 155 (15%) | 58 (98%) | 96 (97%) | | 1 (0.5%) | | 0 (0%) | | 0 (0%) | | 0 (0%) | | 0 (0%) | | 0 (0%) | |  | |  |  |
| General Medicine | 650 (63%) | 0 (0%) | 0 (0%) | | 190 (99%) | | 457 (100%) | | 0 (0%) | | 0 (0%) | | 0 (0%) | | 3 (12%) | |  | |  |  |
| Haematology | 59 (5.7%) | 0 (0%) | 0 (0%) | | 0 (0%) | | 0 (0%) | | 59 (100%) | | 0 (0%) | | 0 (0%) | | 0 (0%) | |  | |  |  |
| Specialized medical unit | 154 (15%) | 1 (1.7%) | 3 (3.0%) | | 0 (0%) | | 0 (0%) | | 0 (0%) | | 72 (100%) | | 78 (100%) | | 0 (0%) | |  | |  |  |
| Undetermined | 21 (2.0%) | 0 (0%) | 0 (0%) | | 0 (0%) | | 0 (0%) | | 0 (0%) | | 0 (0%) | | 0 (0%) | | 21 (88%) | |  | |  |  |
| **Treatment facility type** |  |  |  | |  | |  | |  | |  | |  | |  | | <0.001 | |  |  |
| Non-academic hospital | 274 (26%) | 30 (51%) | 6 (6.1%) | | 119 (62%) | | 43 (9.4%) | | 13 (22%) | | 51 (71%) | | 8 (10%) | | 4 (17%) | |  | |  |  |
| Academic hospital | 765 (74%) | 29 (49%) | 93 (94%) | | 72 (38%) | | 414 (91%) | | 46 (78%) | | 21 (29%) | | 70 (90%) | | 20 (83%) | |  | |  |  |
| **EDI quintile** |  |  |  | |  | |  | |  | |  | |  | |  | | 0.034 | |  |  |
| 1 | 159 (16%) | 5 (8.6%) | 15 (15%) | | 30 (16%) | | 66 (15%) | | 10 (18%) | | 13 (19%) | | 11 (14%) | | 9 (38%) | |  | |  |  |
| 2 | 174 (17%) | 11 (19%) | 22 (22%) | | 24 (13%) | | 83 (18%) | | 5 (9.1%) | | 7 (10%) | | 17 (22%) | | 5 (21%) | |  | |  |  |
| 3 | 224 (22%) | 13 (22%) | 16 (16%) | | 39 (21%) | | 98 (22%) | | 20 (36%) | | 15 (22%) | | 19 (25%) | | 4 (17%) | |  | |  |  |
| 4 | 278 (27%) | 20 (34%) | 35 (35%) | | 52 (27%) | | 119 (26%) | | 14 (25%) | | 18 (26%) | | 18 (23%) | | 2 (8.3%) | |  | |  |  |
| 5 | 190 (19%) | 9 (16%) | 11 (11%) | | 45 (24%) | | 87 (19%) | | 6 (11%) | | 16 (23%) | | 12 (16%) | | 4 (17%) | |  | |  |  |
| Unknown | 14 | 1 | 0 | | 1 | | 4 | | 4 | | 3 | | 1 | | 0 | |  | |  |  |
| **AML sub-type** |  |  |  | |  | |  | |  | |  | |  | |  | | <0.001 | |  |  |
| AML-RCA | 63 (6.1%) | 0 (0%) | 8 (8.1%) | | 2 (1.0%) | | 40 (8.8%) | | 5 (8.5%) | | 1 (1.4%) | | 6 (7.7%) | | 1 (4.2%) | |  | |  |  |
| PML-RARA | 48 (4.6%) | 0 (0%) | 9 (9.1%) | | 2 (1.0%) | | 31 (6.8%) | | 2 (3.4%) | | 0 (0%) | | 3 (3.8%) | | 1 (4.2%) | |  | |  |  |
| AML-MRC | 121 (12%) | 8 (14%) | 8 (8.1%) | | 30 (16%) | | 60 (13%) | | 1 (1.7%) | | 3 (4.2%) | | 10 (13%) | | 1 (4.2%) | |  | |  |  |
| Therapy related AML/MDS | 251 (24%) | 18 (31%) | 12 (12%) | | 42 (22%) | | 67 (15%) | | 41 (69%) | | 40 (56%) | | 22 (28%) | | 9 (38%) | |  | |  |  |
| AML-NOS | 427 (41%) | 12 (20%) | 56 (57%) | | 68 (36%) | | 226 (49%) | | 7 (12%) | | 15 (21%) | | 31 (40%) | | 12 (50%) | |  | |  |  |
| AML with mutated CEBPA/NPM1 | 129 (12%) | 21 (36%) | 6 (6.1%) | | 47 (25%) | | 33 (7.2%) | | 3 (5.1%) | | 13 (18%) | | 6 (7.7%) | | 0 (0%) | |  | |  |  |
|  |  |  |  | |  | |  | |  | |  | |  | |  | |  | |  |  |
|  |  |  |  | |  | |  | |  | |  | |  | |  | |  | |  |  |
| **AML secondary profile** |  |  |  | |  | |  | |  | |  | |  | |  | | <0.001 | |  |  |
| de novo AML | 765 (74%) | 39 (66%) | 85 (86%) | | 145 (76%) | | 379 (83%) | | 15 (25%) | | 32 (44%) | | 55 (71%) | | 15 (62%) | |  | |  |  |
| t-MDS | 149 (14%) | 9 (15%) | 2 (2.0%) | | 20 (10%) | | 33 (7.2%) | | 33 (56%) | | 32 (44%) | | 15 (19%) | | 5 (21%) | |  | |  |  |
| t-AML | 125 (12%) | 11 (19%) | 12 (12%) | | 26 (14%) | | 45 (9.8%) | | 11 (19%) | | 8 (11%) | | 8 (10%) | | 4 (17%) | |  | |  |  |
| **Karyotype/FISH** |  |  |  | |  | |  | |  | |  | |  | |  | | <0.001 | |  |  |
| Karyotype and fish not done | 196 (19%) | 29 (50%) | 6 (6.1%) | | 83 (44%) | | 31 (6.8%) | | 5 (8.6%) | | 31 (43%) | | 8 (10%) | | 3 (14%) | |  | |  |  |
| CARYO or FISH realized | 832 (81%) | 29 (50%) | 92 (94%) | | 104 (56%) | | 424 (93%) | | 53 (91%) | | 41 (57%) | | 70 (90%) | | 19 (86%) | |  | |  |  |
| Unknown | 11 | 1 | 1 | | 4 | | 2 | | 1 | | 0 | | 0 | | 2 | |  | |  |  |
| **Initial cytogenetic prognostic staging** |  |  |  | |  | |  | |  | |  | |  | |  | | <0.001 | |  |  |
| Favourable | 155 (19%) | 2 (7.1%) | 27 (30%) | | 6 (6.0%) | | 100 (24%) | | 5 (9.4%) | | 2 (5.3%) | | 10 (14%) | | 3 (17%) | |  | |  |  |
| Intermediate | 422 (52%) | 18 (64%) | 38 (42%) | | 56 (56%) | | 198 (48%) | | 25 (47%) | | 28 (74%) | | 49 (71%) | | 10 (56%) | |  | |  |  |
| Adverse | 228 (28%) | 8 (29%) | 25 (28%) | | 38 (38%) | | 111 (27%) | | 23 (43%) | | 8 (21%) | | 10 (14%) | | 5 (28%) | |  | |  |  |
| Unknown | 234 | 31 | 9 | | 91 | | 48 | | 6 | | 34 | | 9 | | 6 | |  | |  |  |
| **Charlson comorbidity index** |  |  |  | |  | |  | |  | |  | |  | |  | | <0.001 | |  |  |
| No comorbidities | 489 (48%) | 16 (28%) | 64 (65%) | | 73 (40%) | | 267 (59%) | | 14 (24%) | | 16 (22%) | | 30 (39%) | | 9 (41%) | |  | |  |  |
| Low-mild comorbidities | 360 (35%) | 24 (42%) | 24 (24%) | | 70 (39%) | | 150 (33%) | | 29 (50%) | | 30 (42%) | | 24 (32%) | | 9 (41%) | |  | |  |  |
| Severe comorbidities | 167 (16%) | 17 (30%) | 11 (11%) | | 38 (21%) | | 34 (7.5%) | | 15 (26%) | | 26 (36%) | | 22 (29%) | | 4 (18%) | |  | |  |  |
| Unknown | 23 | 2 | 0 | | 10 | | 6 | | 1 | | 0 | | 2 | | 2 | |  | |  |  |
|  |  |  |  | |  | |  | |  | |  | |  | |  | |  | |  |  |
| **Initial treatment modalities** |  |  |  | |  | |  | |  | |  | |  | |  | | <0.001 | |  |  |
| Untreated/non-curative treatment | 534 (52%) | 53 (91%) | 18 (18%) | | 184 (97%) | | 138 (30%) | | 33 (56%) | | 65 (92%) | | 33 (42%) | | 10 (43%) | |  | |  |  |
| Curative treatment | 499 (48%) | 5 (8.6%) | 80 (82%) | | 6 (3.2%) | | 318 (70%) | | 26 (44%) | | 6 (8.5%) | | 45 (58%) | | 13 (57%) | |  | |  |  |
| Unknown | 6 | 1 | 1 | | 1 | | 1 | | 0 | | 1 | | 0 | | 1 | |  | |  |  |
| **Number of chemotherapy lines** |  |  |  | |  | |  | |  | |  | |  | |  | | <0.001 | |  |  |
| 0 | 1 (0.1%) | 0 (0%) | 0 (0%) | | 1 (1.4%) | | 0 (0%) | | 0 (0%) | | 0 (0%) | | 0 (0%) | | 0 (0%) | |  | |  |  |
| 1 line | 482 (63%) | 20 (95%) | 60 (63%) | | 61 (84%) | | 247 (60%) | | 17 (40%) | | 31 (91%) | | 34 (57%) | | 12 (57%) | |  | |  |  |
| 2 lines | 186 (24%) | 1 (4.8%) | 24 (25%) | | 10 (14%) | | 108 (26%) | | 16 (38%) | | 3 (8.8%) | | 18 (30%) | | 6 (29%) | |  | |  |  |
| >2lines | 91 (12%) | 0 (0%) | 11 (12%) | | 1 (1.4%) | | 59 (14%) | | 9 (21%) | | 0 (0%) | | 8 (13%) | | 3 (14%) | |  | |  |  |
| Unknown | 279 | 38 | 4 | | 118 | | 43 | | 17 | | 38 | | 18 | | 3 | |  | |  |  |
| **Cytological response to first line chemotherapy** | | | |  | |  | |  | |  | |  | |  | |  | | <0.001 | |  |
| Failure | 228 (34%) | 13 (76%) | 21 (24%) | | 42 (82%) | | 95 (25%) | | 17 (44%) | | 17 (65%) | | 15 (28%) | | 8 (44%) | |  | |  |  |
| Partial response/Stable disease | 96 (14%) | 3 (18%) | 3 (3.5%) | | 6 (12%) | | 54 (14%) | | 11 (28%) | | 9 (35%) | | 7 (13%) | | 3 (17%) | |  | |  |  |
| Complete remission | 345 (52%) | 1 (5.9%) | 62 (72%) | | 3 (5.9%) | | 230 (61%) | | 11 (28%) | | 0 (0%) | | 31 (58%) | | 7 (39%) | |  | |  |  |
| Unknown | 370 | 42 | 13 | | 140 | | 78 | | 20 | | 46 | | 25 | | 6 | |  | |  |  |
| **Inclusion in clinical trial** | | | |  | |  | |  | |  | |  | |  | |  | | <0.001 | |  |
| No | 570 (75%) | 22 (100%) | 62 (65%) | | 73 (99%) | | 281 (68%) | | 35 (83%) | | 34 (100%) | | 43 (72%) | | 20 (91%) | |  | |  |  |
| Yes | 193 (25%) | 0 (0%) | 33 (35%) | | 1 (1.4%) | | 133 (32%) | | 7 (17%) | | 0 (0%) | | 17 (28%) | | 2 (9.1%) | |  | |  |  |
| Unknown | 276 | 37 | 4 | | 117 | | 43 | | 17 | | 38 | | 18 | | 2 | |  | |  |  |
| **Treated with immunotherapy** | | | |  | |  | |  | |  | |  | |  | |  | | <0.001 | |  |
| Immunotherapy | 89 (8.6%) | 0 (0%) | 14 (14%) | | 2 (1.0%) | | 52 (11%) | | 5 (8.5%) | | 1 (1.4%) | | 12 (15%) | | 3 (12%) | |  | |  |  |
| No immunotherapy | 950 (91%) | 59 (100%) | 85 (86%) | | 189 (99%) | | 405 (89%) | | 54 (92%) | | 71 (99%) | | 66 (85%) | | 21 (88%) | |  | |  |  |
| **Chemotherapy ±HSCT** | | | |  | |  | |  | |  | |  | |  | |  | | <0.001 | |  |
| Chemotherapy+ HSCT | 184 (18%) | 0 (0%) | 28 (29%) | | 0 (0%) | | 133 (29%) | | 7 (12%) | | 0 (0%) | | 15 (19%) | | 1 (4.3%) | |  | |  |  |
| Chemotherapy only | 463 (45%) | 10 (17%) | 63 (64%) | | 43 (23%) | | 255 (56%) | | 26 (44%) | | 10 (14%) | | 39 (50%) | | 17 (74%) | |  | |  |  |
| Untreated | 386 (37%) | 48 (83%) | 7 (7.1%) | | 147 (77%) | | 68 (15%) | | 26 (44%) | | 61 (86%) | | 24 (31%) | | 5 (22%) | |  | |  |  |
| Unknown | 6 | 1 | 1 | | 1 | | 1 | | 0 | | 1 | | 0 | | 1 | |  | |  |  |
| ^1^n (%); Median (IQR) | | | | | | | | | | | | | | | | | | | | |
| ^2^Fisher's Exact Test for Count Data with simulated p-value (based on 2000 replicates); Kruskal-Wallis rank sum test | | | | | | | | | | | | | | | | | | | | |
